# A Novel Clustering Method Based on Adjacent Grids Searching

**DOI:** 10.3390/e25091342

**Published:** 2023-09-15

**Authors:** Zhimeng Li, Wen Zhong, Weiwen Liao, Jian Zhao, Ming Yu, Gaiyun He

**Affiliations:** 1 School of Control and Mechanical Engineering, Tianjin Chengjian University, Tianjin 300384, China; lzmcxg@tcu.edu.cn (Z.L.);; 2 School of Computer and Information Engineering, Tianjin Chengjian University, Tianjin 300384, China; 3 School of Mechanical Engineering, Tianjin University, Tianjin 300072, China

**Keywords:** unsupervised learning, clustering, grid-based method, high dimension, large scale, denoise

## Abstract

Clustering is used to analyze the intrinsic structure of a dataset based on the similarity of datapoints. Its widespread use, from image segmentation to object recognition and information retrieval, requires great robustness in the clustering process. In this paper, a novel clustering method based on adjacent grid searching (CAGS) is proposed. The CAGS consists of two steps: a strategy based on adaptive grid-space construction and a clustering strategy based on adjacent grid searching. In the first step, a multidimensional grid space is constructed to provide a quantization structure of the input dataset. The noise and cluster halo are automatically distinguished according to grid density. Moreover, the adaptive grid generating process solves the common problem of grid clustering, in which the number of cells increases sharply with the dimension. In the second step, a two-stage traversal process is conducted to accomplish the cluster recognition. The cluster cores with arbitrary shapes can be found by concealing the halo points. As a result, the number of clusters will be easily identified by CAGS. Therefore, CAGS has the potential to be widely used for clustering datasets with different characteristics. We test the clustering performance of CAGS through six different types of datasets: dataset with noise, large-scale dataset, high-dimensional dataset, dataset with arbitrary shapes, dataset with large differences in density between classes, and dataset with high overlap between classes. Experimental results show that CAGS, which performed best on 10 out of 11 tests, outperforms the state-of-the-art clustering methods in all the above datasets.

## 1. Introduction

As an unsupervised learning technique, clustering is widely used to explore the structure of a given dataset [1,2,3]. Due to the growth of the Internet of Things, data is generated every day across the globe [2]. Clustering techniques are required to unravel important hidden facts and understand the massive data. In addition, clustering could also be used in particular domains, such as gene expression profiles, where domain experts often provide incomplete knowledge in the form of pairwise constraints [3].

Based on different strategies, the clustering methods can be mainly classified into partition-based clustering, density-based clustering, hierarchical clustering and grid-based clustering. The above clustering methods are usually offline, which needs to repeat the whole clustering process when new data arrives. Partition-based clustering [4,5] assigns a set of data points to *k* clusters by optimizing a criterion function, where k is the number of clusters as an input parameter. The common problem with partition-based clustering is that only hyperspherical-shaped clusters can be found by this kind of method. Compared with partition-based clustering, density-based clustering [6] can find arbitrarily shaped clusters in a spatial dataset. However, density-based clustering performs poorly for low signal to noise Ratio (SNR) datasets and high dimensional datasets. Hierarchical clustering [7,8,9,10] is another kind of clustering strategy that groups data through a sequence of nested partitions. This kind of method can identify the nodes sparsely distributed in the dataset called outliers. However, it is inefficient to handle the noise points with uniform distribution. A clustering method called clustering by fast search and find of density peaks (CFSFDP) has been proposed [11] that outperforms the above clustering methods on most datasets in terms of clustering accuracy. However, the number of clusters could not be determined by CFSFDP, and good performance depends on the manual selection of cluster centers through the decision graph. There are a kind of clustering strategies that focus on data transformation and use other clustering methods as the partition part, such as RDNNBMF [12], DGSCF [13], and spectral clustering [14]. RDNNBMF is an algorithm that includes a multilayered structure. Its objective function contains the regularization constraint term on the basis images matrix, which is helpful for classification. The original samples are projected into a high dimensional space by a nonlinear map to adapt to more complex data. DGSCF is a dual graph-regularized sparse concept factorization algorithm. It adopts an optimization framework that enhances the ability of feature selection and sparsity to eliminate the influence of noise factors on the algorithm’s performance. Spectral clustering is proposed to solve the problem of partition-based clustering in dealing with datasets with arbitrarily shaped distributions. To complete the clustering process, a *k*-means clustering algorithm should be performed at the end of this kind of method.

All of the above clustering methods deal directly with data points one by one. As a result, the time complexity is at least O(*N*), and thus, they are limited to processing large-scale datasets. This problem can be solved by grid-based clustering methods [15,16,17,18,19,20] because the number of grids is independent of the scale of the dataset. Representative grid-based clustering algorithms include GRIDCLUS [15], STING [16], WaveCluster [17], CLIQUE [18], optimal grid-clustering [19], and GDILC [20]. Despite the high efficiency of grid-based methods in processing large-scale data points, some drawbacks also exist in these algorithms. GRIDCLUS and STING could not provide high clustering accuracy when clustering datasets with arbitrary shapes. It is difficult for WaveCluster to be used for datasets with three or higher dimensions. CLIQUE and optimal grid-clustering are specially designed for high-dimension datasets. However, CLIQUE could only partition the dataset in which the clusters do not overlap with each other, and optimal grid-clustering could only find the hyperspherical clusters. GDILC needs the information of each data point to construct the grid space, which greatly reduces algorithm efficiency.

In summary, it is very difficult for a clustering method to possess the following properties simultaneously: (1) the ability to cluster on datasets with noise; (2) the ability to cluster on large-scale datasets; (3) the ability to cluster on high-dimensional datasets; (4) the ability of clustering on datasets with arbitrary shapes; (5) the ability of clustering on datasets with large density contrast; (6) the ability of clustering on datasets with high-overlap between classes. In this paper, we propose a clustering based on adjacent grid searching (CAGS) to address the above challenges. In the CAGS, an adaptive multidimensional grid generation method is established, which makes CAGS effectively achieve clustering on large-scale datasets. A density-based noise threshold is used to handle both the outliers and noise points contained in the dataset. A density-based halo threshold is applied to identify boundary points whose densities are lower than those of center points of clusters, by which clustering on the dataset with high overlap between classes can be accomplished. Clustering principles based on adjacent grid operators and adjacent grid databases are proposed to deal with low-dimensional and high-dimensional datasets, respectively. In the recursive process of grid clustering, the algorithm can automatically detect the number of clusters and find clusters with arbitrary shapes.

The major contributions of this paper can be described as follows.

1. We propose a novel grid-based clustering method that shows broad robustness in clustering the above six types of datasets.
2. The proposed CAGS could automatically identify the number of clusters and detect the center of each cluster.
3. We assign some key attributes, such as density, to cells in grid space so that the cluster center can be found.
4. In addition to randomly generated cluster labels, CAGS also outputs further intrinsic information about the dataset, such as cluster density. This intrinsic information could be used to indicate the real-world properties of each cluster.

The remainder of this paper is organized as follows. Section 2 presents related papers. Section 3 provides the details of the CAGS clustering algorithm. To demonstrate the validity of CAGS, comprehensive experiments on the international standard dataset and the proposed synthetic dataset are performed in Section 4. Finally, some useful conclusions of this paper are given in Section 5.

## 2. Related Work

Recently, some new grid-based clustering methods have been proposed to solve the above problems [19,20,21,22,23,24]. These methods captured attention with the advantage over other approaches because they process data with grid cells. Most grid-based methods perform clustering through several main steps, such as grid space construction, grid cell preprocess, and cluster generation. Grid space is generally composed of nodes, lines, and cells. To the best of our knowledge, existing grid clustering algorithms work by dealing with nodes and cells. The former mainly includes FDGB [21] and GCBD [22], while the latter mainly includes GBCN [23], GCDPP [24], NGCGAL [25], and CMSPGD [26]. However, different grid-based clustering methods have their own considerations in grid space, node or cell processing, and cluster generation strategies, resulting in differences in clustering performance.

FDGB adopted a fuzzy-type membership function to define the relationship between data points and nodes. In the grid space of FDGB, the raw data points were assigned to neighbor nodes based on different weights. Then, the clusters could be found through the method called finding mountain ridges. Some instances were given in two-dimensional situations to verify the effectiveness of the algorithm. Different from FDGB, GCBD first divided nodes into core nodes and boundary nodes by using a specific cut-off value. The cluster-finding process was implemented through the connection strategy. The advantage is that it can handle halo data points in the area where clusters come into contact. However, from a geometric perspective, the number of nodes in a cell will increase exponentially with dimensionality. As a result, computational costs will become unacceptable for clustering high-dimensional datasets.

For algorithms that use cells as processing objects, the above problem does not exist. GBCN provided a simple loop to construct each cluster by traversing the cells with non-empty cells around themselves. It does not distinguish the rank of cells, so it cannot handle clusters with overlapping regions. GCDPP counted the number of location points in each grid and used it as grid density. Then, the discrete wavelet transform was employed so as to classify the matrix formed via the grids’ density. Grids of different levels are merged according to neighborhood similarity to form the final clusters. This method is very similar to WaveCluster and will encounter difficulties when processing high-dimensional datasets.

Moreover, many grid-based clustering methods are proposed to solve problems in a specific domain. NGCGAL is a grid clustering algorithm specifically designed for wireless sensor localization, focusing on the integration of IoT and WSN for real-time localization systems. CMSPGD is a clustering algorithm based on stay points and grid density that can be used to extract urban hotspot areas from GPS data. Both methods limit the input to two-dimensional datasets.

## 3. Principle of CAGS

In CAGS, data points in the same cell are considered members of the same cluster. Then, two key parts of CAGS to ensure the effectiveness and robustness of our algorithm are constructed. The first is an adaptive grid-space constructing strategy that fits both the large-scale dataset and the high-dimensional dataset. The second is a clustering strategy based on adjacent grid searching, which can find clusters with arbitrary shapes by processing cells in the adaptive grid space.

### 3.1. Construction of Adaptive Grid Space

**Definition** **1.** *Given a dataset with* N *instances that each instance has d attributes, we express it as a multidimensional dataset* $D^{d}$.

(1) $$D^{d}={\left\{X_{1}^{d},X_{2}^{d},\cdots ,X_{N}^{d}\right\}}^{T}$$

where $X_{i}^{d}$ is the $i^{th}$ instance of $D^{d}$.

(2) $$X_{i}^{d}=\left\langle x_{i}^{d}(1),x_{i}^{d}(2),\cdots ,x_{i}^{d}(d)\right\rangle$$

The multidimensional dataset is processed in a multidimensional finite space with d orthogonal continuous coordinates, which can be defined as

(3) $$S=\left\{S_{1},S_{2},\cdots ,S_{d}\right\}$$

where $S_{i}$ denotes the $i^{th}$ coordinate of the space. It can be constituted by a limited number of right open intervals by taking the min and max value of a coordinate and then dividing it into *R* intervals of the same length. The $j^{th}$ interval of the $i^{th}$ dimension can be defined as

(4) $$u_{ij}=\left\{\begin{array}{c} {}[{sc}_{i}(j-1),{sc}_{i}(j))\;\;\;\;j<R\\ {}[{sc}_{i}(j-1),{sc}_{i}(j)]\;\;\;\;j=R \end{array}\right.$$

where $sc_{i}^{D}(j-1)$ and $sc_{i}^{D}(j)$ are the left and right boundaries of $u_{ij}$, respectively. Thus, we have

(5) $$S_{i}=\underset{j\in Z}{\cup }\left\{u_{ij}\right\}$$

**Definition** **2.** *In the* $i^{th}$ *dimension of the multidimensional finite space, a coordinate sequence* $SC_{i}^{d}$ *is used to divide* S*, which can be expressed as*

(6) $$SC_{i}^{d}=\left\{sc_{i}^{d}(0),sc_{i}^{d}(1),\cdots ,sc_{i}^{d}(R)\right\}$$

**Definition** **3.** *To mesh the input data into hyperrectangle cells, an adaptive grid space* $G^{d}$ *is constructed by using* $SC_{i}^{d}$.

(7) $$G^{d}=\left\{C_{1}^{d},C_{2}^{d},\cdots ,C_{M}^{d}\right\}$$

where $C_{i}^{d}$ is the $i^{th}$ cell of the grid space, $M=R^{d}$ is the number of cells in the grid space. For each cell, 3 properties are set to connect the grid space and the dataset.

(8) $$C_{i}^{D}=\left\{location,member,density\right\}$$

The property *location* records the coordinate information of the cell, the *member* records all data points contained in the cell, and *density* records the number of data points in a unit volume. The *location* of $C_{i}^{D}$ can be expressed as

(9) $$location(C_{i}^{D})=\left\langle c_{i1},c_{i2},\cdots ,c_{id}\right\rangle$$

where the subscript i can be calculated by

(10) $$i=\sum_{j=1}^{d}[(c_{ij}-1)R^{j-1}]+1$$

Using Equations (2), (6) and (9), all data points in $D^{d}$ can be assigned to their grid cells. When constructing a grid space, cell size has a significant impact on clustering performance. A very large cell size will lead to insufficient cells to partition data points from different clusters. On the contrary, a small cell size will lead to so many cells that the *density* of each cell is too low. This will reduce the accuracy and efficiency of clustering. Furthermore, the number of cells increases exponentially with the dimension of the dataset, resulting in the curse of dimensionality. To solve the above problems, an adaptive grid-space generation strategy is proposed in the CAGS. Firstly, the resolution R is determined according to the scale of the input dataset by the following formula

(11) $$R=\mathrm{Int}(\sqrt[{d}]{N}\cdot f_{R})+1$$

where Int(x) denotes the forward rounding function, N is the number of data points, $f_{R}$ is the resolution coefficient. Secondly, in our algorithm, the grid space can be efficiently constructed by scanning all data points at once. For a data point, it will be checked if it belongs to any existing cell. If so, update the properties of this cell; otherwise, create a new cell. It is noted that the grid space obtained by using our method is very economical since only grid cells containing data points are recorded. Therefore, CAGS can effectively deal with a high-dimensional clustering problem because a bulk of null cells are removed in the grid space. However, the cell number of the null cells is reserved to reactivate them if necessary. The pseudocode for the construction of adaptive grid space is listed in Algorithm 1.
**Algorithm 1:** Pseudocode for construction of adaptive grid space.                       **Input:** dataset $D^{d}$ and resolution R and coordinate sequence $SC_{i}^{D}$.                       **Output:** Multidimensional grid space $G^{D}$.1: Begin2: $G^{D}\leftarrow \phi$3: N← samples number of $D^{d}$4: d← dimension of $D^{d}$5: for k = 1 to N do6:    cellnum←07:    for i = 1 to d do8:      for j = 1 to R do9:        if ${sc}_{i}^{D}(j-1)\leq x_{k}^{d}(i)<{sc}_{i}^{D}(j)$ then10:          $cellnum\leftarrow cellnum+(j-1)R^{j-1}$11:          $c\left(i\right)\leftarrow j$12:    $location\left(C_{cellnum}^{D}\right)\leftarrow \langle c\left(1\right),c\left(2\right),\cdots ,c(d)\rangle$13:    $mumber\left(C_{cellnum}^{D}\right)\leftarrow mumber\left(C_{cellnum}^{D}\right){\cup X}_{k}^{d}$14:    $density\left(C_{cellnum}^{D}\right)\leftarrow density\left(C_{cellnum}^{D}\right)+1$15: if $C_{cellnum}^{D}\notin G^{D}$ then16: $G^{D}\leftarrow G^{D}\cup C_{cellnum}^{D}$

**Figure 1:**
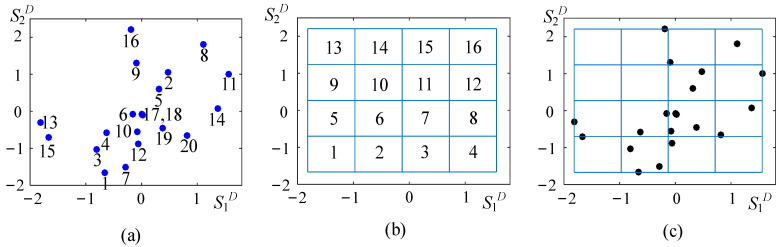
Example of multidimensional grid space. (**a**) 2-dimensional Gaussian dataset; (**b**) cell numbering in uniform grid spaces; (**c**) partitioning in uniform grid space.

The construction process of adaptive grid space will be demonstrated using a 2-dimensional dataset, as shown in Figure 1. The dataset contains 16 instances conforming to Gaussian distributions, which are marked from 1 to 16 in Figure 1a. Then, the spatial extent of the dataset is divided into a 4 × 4 grid through Algorithm 1 when the resolution coefficient $f_{R}$ is set as 0.8, as shown in Figure 1b. Each cell has a different number of instances in Figure 1c, and there are 4 empty cells. The meshing results of the 2-dimensional dataset, cell number, location, density, and members of each nonnull cell, are listed in Table 1.

### 3.2. Clustering Strategy Based on Adjacent Grids Searching

The clustering is accomplished by using adjacent grid searching. In the grid space $G^{D}$, the adjacent cells of a cell $C_{i}^{D}$ is defined as $AC_{i}^{D}$ whose locations are

(12) $$location(AC_{i}^{D})=location(C_{i}^{D})+Aopt^{d}$$

**Figure 2:**
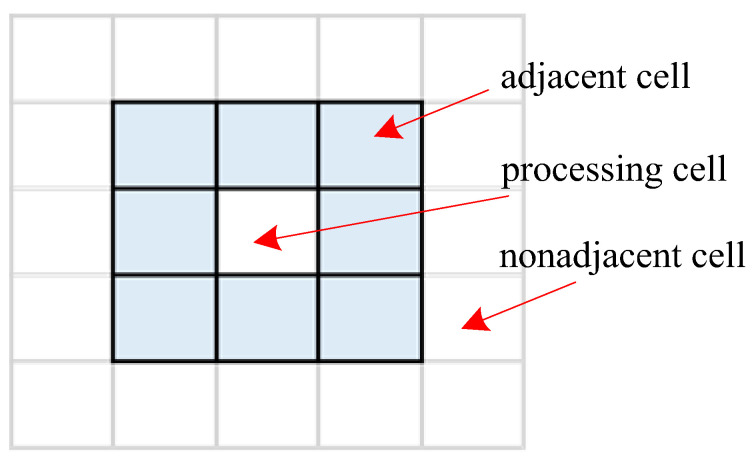
Diagram of the 2-dimension adjacent operator.

**Definition** **4.** *In Equation (12),* $Aopt^{d}$ *is a d-dimension adjacent operator that can be defined as*

(13) $$Aopt^{d}=\left\{L_{1}^{d},L_{2}^{d},\cdots ,L_{T}^{d}\right\}-0$$

where $L_{i}$ is the $i^{th}$ coordinate vector ascending as ternary notation, **0** denotes the zero vector. For example, the adjacent operator in the 2-dimension grid space, as shown in Figure 2, can be written as

(14) $$Aopt^{2}=\left\{\left\langle -1,1\right\rangle ,\left\langle -1,0\right\rangle ,\left\langle -1,1\right\rangle ,\left\langle 0,-1\right\rangle ,\left\langle 0,1\right\rangle ,\left\langle 1,-1\right\rangle ,\left\langle 1,0\right\rangle ,\left\langle 1,1\right\rangle \right\}$$

The clustering process includes two stages called core cell traversal and peripheral cell clustering, as shown in Algorithm 2. In the first stage, the core cell traversal starts with the densest cell of unlabeled cells, and a new cluster is established. Meanwhile, the cell with the highest density is labeled as the center of the cluster. The algorithm then seeks the nonnull cell from the adjacent cells of each cell in the cluster and adds it to the current cluster until the cluster cannot be expanded. At the end of this stage, each core cell will be assigned a label corresponding to a cluster. In the second stage, peripheral cells will be distributed to clusters established in the first stage. For a peripheral cell, the algorithm finds the nonnull cells from its adjacent cells and then distributes them to the cluster of the nearest adjacent cell. In particular, if a peripheral cell does not have nonnull cells from its adjacent cells, the peripheral cell will be defined as a new cluster. To find the nearest adjacent cell of a peripheral cell, the distance of two cells is the distance of their centers, which can be calculated as follows:

(15) $$centre(C_{i}^{D})=\frac{\sum_{j=1}^{n}X_{j}^{D}}{n}$$

where *n* is the point number of the cell $C_{i}^{D}$. $X_{j}^{D}$ is the *i*th instance of the multidimensional dataset defined in Equation (2).
**Algorithm 2:** Pseudocode for low-dimensional clustering based on adjacent grid searching.                      **Input:**$\;\mathrm{dataset}\;D^{d}$, noise cells *NC*, peripheral cells *PC*, core cells *CC*                      **Output:** cluster label, cluster density, cluster number *m*1: Initialization;2: Find the adjacent cells for each nonnull cell in *CC* according to Equation (12);3: Rank all cells in descending order of *density*;4: CM← cells number of CC, m←0;5: **For** *i* = 1 to *CM* **Do**6:    **If** the *i*^th^ cell in *CC* is not handled, **Then**7:       m← *m* +1, tempCluster←$\mathrm{the}\;i^{\mathrm{th}}\;\mathrm{cell}\;\mathrm{in}\;\mathrm{CC},\;j\leftarrow$ 1;8:       **While** not all cells in *tempCluster* are handled, **Do**9:          Label the *j*^th^ cell in *tempCluster* to be the *m*^th^ cluster;10:        Add the adjacent cells in *CC* of the *j*^th^ cell to *tempCluster*;11:        j← *j* + 1;12:    **end While**13: **end If**14: **end For**15: PM← cells number of *PC*;16: **While** not all cells in *PC* are handled, **Do**17: **For** *i* = 1 to *PM* **Do**18:    **If** the *i*^th^ cell in *PC* is not handled, **Then**19:      Find its adjacent cells according to Equation (12);20:      Select the above adjacent cells which are in *CC*;21:      Calculate the distance between the cell and its adjacent cells in *CC* according to                Equation (15);22:      Label the *i*th cell in *PC* to be the same cluster with its nearest adjacent cell in *CC*;23:    **end If**24: **end For**25: **end While**26: Label the data points according to their cells;27: Calculate the mean density of the cells of each cluster;

When clustering a dataset with 5 or more dimensions, the adjacent operator is not sufficient to find the adjacent cells. Therefore, the adjacent cells of $C_{i}^{D}$ are redefined by constructing the *k*-adjacent vector of each cell in the grid in the space $G^{D}$. Here, the *k*-adjacent vector of the cell $C_{i}^{D}$ can be found by a threshold as follows

(16) $$threshold=\sqrt{k}$$

That is, $C_{j}^{D}$ is defined as the *k*-adjacent cell of $C_{i}^{D}$ if the distance between $C_{i}^{D}$ and $C_{j}^{D}$ is less than the threshold. In this paper, the default value of *k* is 2. Then, the clustering process could be accomplished by Algorithm 3 instead of Algorithm 2.
**Algorithm 3:** Pseudocode for high-dimensional clustering based on *k*-adjacent cells searching.                      **Input:**$\;\mathrm{dataset}\;D^{d}$, multidimensional grid space $G^{d}$                      **Output:** cluster label, cluster density, cluster number *m*1: Initialization; 2: Rank all cells in descending order of *density*; 3: M← $\mathrm{cells}\;\mathrm{number}\;\mathrm{of}\;G^{d}$; 4: Traverse all grids; for the *i*^th^ cell, construct its *k*-adjacent vector from the (*i* + 1)^th^ to *M*^th^    cells according to Equation (16); 5: m← 0 6: **For** *i* = 1 to *M* **Do** 7:    **If** the *i*th cell exists, **Then** 8:      m← *m* + 1; 9:      Label the *i*^th^ cell to be the *m*^th^ cluster; 10:    Find the *k*-adjacent cells of the *i*^th^ cell; 11:    Label these *k*-adjacent cells to be the *m*^th^ cluster; 12:    Delete these *k*-adjacent cells and the current cell; 13: **end If** 14: **end For** 15: Label the data points according to their cells; 16: Calculate the mean density of the cells of each cluster;

According to Algorithms 1 and 2, the proposed algorithm is efficient with time complexity less than O(*M*^2^), where *M* is the number of cells. The relationship between *M* and *N* depends on the distribution density of the dataset. In most engineering applications, the distribution density increases with the scale of the dataset. Therefore, the time complexity of CAGS decreases with the number of data points. As a rule of thumb, *M* is usually less than $\sqrt{N}$, thus, the time complexity of CAGS is less than O(*N*).

### 3.3. Selection and Calculation of the Input Parameters

In our method, the noise points could be distinguished by using a noise threshold

(17) $$threN=\frac{\sum_{i=1}^{M_{1}}density(C_{i}^{D})}{M_{1}}f_{N}$$

where $f_{N}$ is the noise coefficient, $M_{1}$ is the number of nonnull cells. The cells whose density is smaller than *threN* will be defined as noise cells and not considered in the next step.

To identify overlap between two adjacent clusters, a halo threshold is proposed to divide the cells into peripheral cells and core cells. If the density of a cell is smaller than the halo threshold, it will be considered a peripheral cell; otherwise, it will be considered a core cell. The halo threshold can be calculated as

(18) $$threH=\frac{\sum_{i=1}^{M_{2}}density(C_{i}^{D})}{M_{2}}f_{H}$$

where $f_{H}$ is the halo coefficient, $M_{2}$ is the total number of nonnull cells after denoising.

In CAGS, the number of clusters could be automatically recognized so that it is not required as an input parameter. However, three essential input parameters need to be determined before clustering. The first one is the resolution coefficient $f_{R}$ which determines the level of detail of the grid space. The larger $f_{R}$ results from the denser grid space, which means that more details of the dataset can be discovered. However, a very large $f_{R}$ may lead to a decrease in clustering efficiency and fragmentation of clusters. The $f_{R}$ is usually set from 0.3 to 3. The second parameter called the noise coefficient $f_{N}$, needs to be set according to the noise level of the dataset. The threshold to define the noise data is adapted to the density distribution of the dataset so that the selection of $f_{N}$ is insensitive to the dense level of the dataset. If a larger $f_{N}$ is given, data points with higher noise levels will be detected, and vice versa. The $f_{N}$ is usually set from 0 to 1.5. The third parameter named the halo coefficient $f_{H}$ is used to divide the cells into peripheral cells and core cells. If the clusters of a dataset have no overlap, the $f_{H}$ can be set to 0, which means no peripheral cells will be defined. The $f_{H}$ is usually set from 0 to 3.

**Figure 3:**
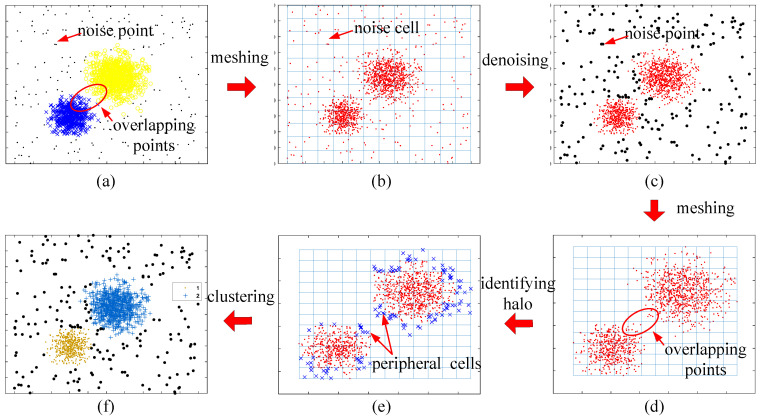
Visualization of clustering process using CAGS (**a**) raw data; (**b**) raw data in the grid space; (**c**) data after denoise; (**d**) denoised data in the new grid space; (**e**) denoised data after identifying halo; (**f**) clustering result.

**Figure 4:**
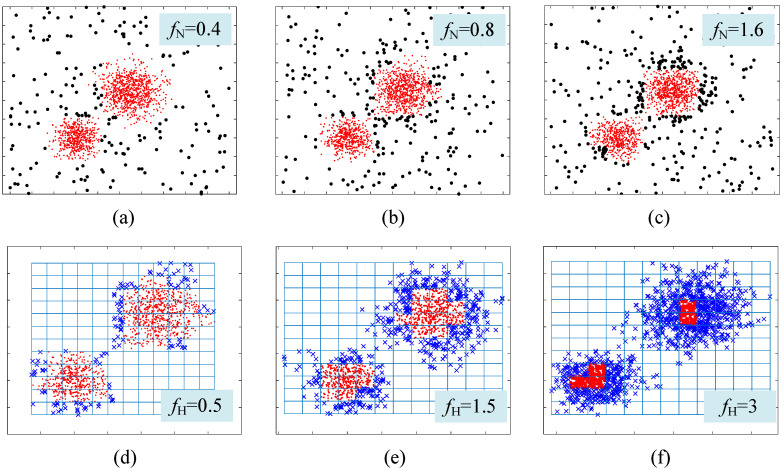
Influence of the noise coefficient and halo coefficient (**a**) *f*_N_ = 0.4; (**b**) *f*_N_ = 0.8; (**c**) *f*_N_ = 1.6; (**d**) *f*_H_ = 0.4; (**e**) *f*_H_ = 1.5; (**f**) *f*_H_ = 3.

The visualization of the clustering process using CAGS and the influence of the noise coefficient and halo coefficient are shown in Figure 3 and Figure 4. In Figure 3a, we can see that two classes of data points overlap with each other, as well as many noise points exist in the background. The data points are first put in the grid space, as shown in Figure 3b. In our method, noise points can be easily detected through the distribution density that is recorded in the cells. When *f*_N_ is set to 0.4, the noise points are well identified in Figure 3c. Then, the meshing process is reused to the denoised data, and the new grid space is constructed. A traversal strategy is adopted to find clusters with arbitrary shapes rather than the iterative optimization strategy, such as that of *k*-means. However, overlapping parts will bring challenges to clustering because the traversal strategy establishes undifferentiated connectivity. That is, it is contradictory to simultaneously discover the clusters with arbitrary shapes that overlap with each other. Therefore, we use the distribution density again to identify the halo part of clusters. This is effective because overlapping parts between clusters, namely the edges of clusters, often have a lower distribution density than that of the cores of the clusters. When *f*_H_ is set to 0.5, the halo points are well identified in Figure 3e. In most cases, small changes in the noise coefficient and halo coefficient have little impact on clustering results, as can be demonstrated in Figure 4. The larger the *f*_N_, the more noise points are identified. However, it can be seen from Figure 4a–c that the main parts of the two clusters have been preserved even if *f*_N_ increases from 0.4 to 1.6. In addition, identifying more or fewer halo cells does not change the clustering results, as the cluster centers are successfully identified, as shown in Figure 4d,e.

An optional input parameter called the merger coefficient $f_{M}$ is proposed for the optimization of the clustering results. The purpose of clustering result optimization is to merge the unnecessary small clusters into the main clusters. The $f_{M}$ could be set to 0 if the clustering results need not be optimized. For a given cluster, the cluster scale is defined as the number of cells, which can be written as scale(k). If this value is lower than the given threshold, the corresponding cluster is considered an unnecessary, small cluster. The scale threshold can be calculated as follows

(19) $$threM=f_{M}(scale_{max}-{scale}_{min})$$

where $f_{M}$ is the merger coefficient.

## 4. Performance Evaluation

### 4.1. Datasets

As mentioned in the introduction, several typical problems have a significant impact on performance in the clustering of most real-world datasets, so CAGS will be tested on 6 different types of datasets, as shown in Table 2. Most of these datasets, with the exception of Test V and Test X, are selected from the benchmark datasets and renowned references. In Test V, we propose a type of synthetic high-dimensional dataset with a dense distribution where clusters can be found in the same subspace. In Test X, we set the distribution of clusters as a Gaussian distribution, which is very common in the real world. The distribution density of adjacent clusters is quite different, which leads to a disturbance from the higher-density cluster to the lower-density cluster in the clustering process. In this section, a series of experiments are staged to study the performance of CAGS. All experiments were run on a PC with a 2.40 GHz processor and 4 GB RAM. 

When ground truth is available, the external clustering evaluation provides more reliable results than the internal clustering evaluation by comparing cluster labels with the class labels. In this paper, five external clustering evaluation indicators are adopted as follows:

(1) Purity (*PUR*) [27]
  (20) $$PUR=\frac{1}{n}\sum_{k=1}^{K}\underset{1\leq k*\leq K*}{\max}\;n_{k,k*}$$
(2) Cluster similarity measure (*CSM*) [28]
  (21) $$CSM=\frac{1}{n}\sum_{k=1}^{K}\underset{1\leq k*\leq K*}{\max}\;\frac{2n_{k,k*}}{n_{k}+n_{k*}}$$
(3) Normalized mutual information (*NMI*) [29]
  (22) $$NMI=\frac{\sum_{k=1}^{K}\sum_{k*=1}^{K*}n_{k,k*}\log\left(n\cdot n_{k,k*}/n_{k}/n_{k*}\right)}{\sqrt{\left(\sum_{k=1}^{K}n_{k}\log\left(n_{k}/n\right)\right)\left(\sum_{k*=1}^{K*}n_{k*}\log\left(n_{k*}/n\right)\right)}}$$
(4) Cluster-based cross entropy (*CluCE*) [27]
  (23) $$CluCE=-\frac{1}{\log K}\sum_{k*=1}^{K*}\frac{n_{k*}}{n}\;\sum_{k=1}^{K}\frac{n_{k,k*}}{n_{k*}}\log\frac{n_{k,k*}}{n_{k*}}$$
(5) Class-based cross entropy (*ClaCE*) [27]

(24) $$ClaCE=-\frac{1}{\log K*}\sum_{k=1}^{K}\frac{n_{k}}{n}\;\sum_{k=1}^{K*}\frac{n_{k,k*}}{n_{k}}\log\frac{n_{k,k*}}{n_{k}}$$

where *n* is the number of data points, *n_k_* and n_k__*_ denote the number of data points in class *k* and cluster k*, *n_k_*_,k*_ denotes the number of data points in class *k* as well as in cluster k*. The performance indexes *PUR*, *CSM*, and *NMI* are used to measure the effectiveness of clustering. It illustrates a better clustering quality when the clustering result shows higher *PUR*, *CSM*, and *NMI*. If *n_k_*_,k*_ = *n_k_* = n_k__*_, their scores will reach 1, indicating a perfect match between the ground truth and clustering results. Conversely, they approach 0.

**Table 2:** Test data of 6 different types of datasets.

Symbol	Data Set	Description
Test Ⅰ	Synthetic point distributions with different levels of white noise	The synthetic datasets with different levels of white noise are proposed in clustering by fast search and finding of density peaks [11].
Test Ⅱ	Large-scale datasets	The large-scale datasets are proposed in BIRCH, an efficient data clustering method for very large databases [7].
Test Ⅲ	Wine	The dataset wine is selected from the benchmark datasets of the UCI machine learning repository [30].
Test Ⅳ	Grammatical facial expression	The dataset grammatical facial expression is proposed in grammatical facial expression recognition with machine learning [31].
Test V	Synthetic high dimensional dense datasets	The dataset has *d* + 1 clusters in an orthogonal space with *d* dimensions. The $i^{th}$ cluster contains $N_{i}$ points with Gaussian distribution around a center point whose $i^{th}$ coordinate is 1, and the other coordinates are 0. In particular, all coordinates of the center point of the 0th cluster are 0.
Test Ⅵ	Flame	The dataset flame is proposed in FLAME, a novel fuzzy clustering method for the analysis of DNA microarray data [32].
Test Ⅶ	3-spiral	The dataset 3-spiral is proposed in robust path-based spectral clustering [33].
Test Ⅷ	Jain	The dataset Jain is proposed in Data Clustering: A User’s Dilemma [34].
Test Ⅸ	Sticks	The dataset sticks are proposed in robust path-based spectral clustering [33].
Test X	Synthetic point distributions with large density contrast	The dataset contains 4 clusters which have 100, 50, 200, and 5000 points from bottom left to top right, respectively. The largest density contrast is nearly 25 times.
Test Ⅺ	Data set S3	This dataset is proposed in the iterative shrinking method for clustering problems [35].

To make a valid comparison, we choose the best result of each algorithm under many different input parameters in each test. All the parameters of the clustering algorithms are classified into three types. The first type of parameters can correspond to the real world, such as the actual number of clusters in *k*-means and CFSFDP. For this type of parameter, the proper values are adopted. The second type of parameter has a straightforward meaning that corresponds to the clustering model, such as *num_cells* in WaveCluster, which denotes the number of cells per dimension in the grid space. For this type of parameter, a wide range of values are adopted to find the optimal clustering results. The standard for determining boundary values is that as the parameter values increase (or decrease), the clustering results continue to deteriorate. The third type of parameters are some key variables in the clustering process that usually have complicated meanings, such as *weights* in WaveCluster and eps in DBSCAN. For this type of parameter, the default values recommended by algorithms are used.

### 4.2. Clustering Datasets with Noise

**Figure 5:**
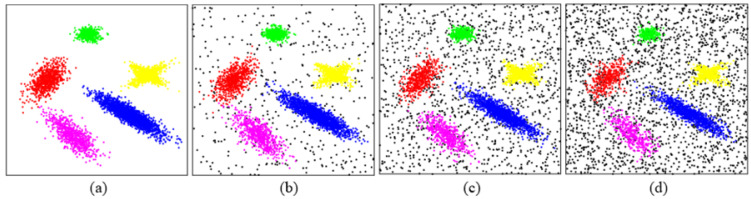
Clustering results for synthetic datasets with noise. (**a**–**d**) Point distributions for samples of 5000 points and the SNR are 100%, 90%, 70%, and 50%, respectively.

Most datasets in the real world contain noise, so the ability to process noise data will greatly improve the recognition accuracy of the clustering algorithm. Synthetic datasets with different levels of white noise (Figure 5) proposed in [11] are used to test the performance of clustering datasets with noise. In this test, SNR is defined as the ratio of non-noise points to all points. Table 3 shows the best-performing values of input parameters selected for the algorithms in Test Ⅰ. Table 4 shows the clustering results of *k*-means, DBSCAN, CFSFDP, WaveCluster, FDGB, and CAGS on Test Ⅰ. The best score of the indicator is marked in bold, and the worst score is marked in italics. It can be seen that CAGS almost outperforms other algorithms at all levels of noise. Algorithms *k*-means, WaveCluster, and FDGB are significantly affected by noisy data.

### 4.3. Clustering Large-Scale Datasets

**Figure 6:**
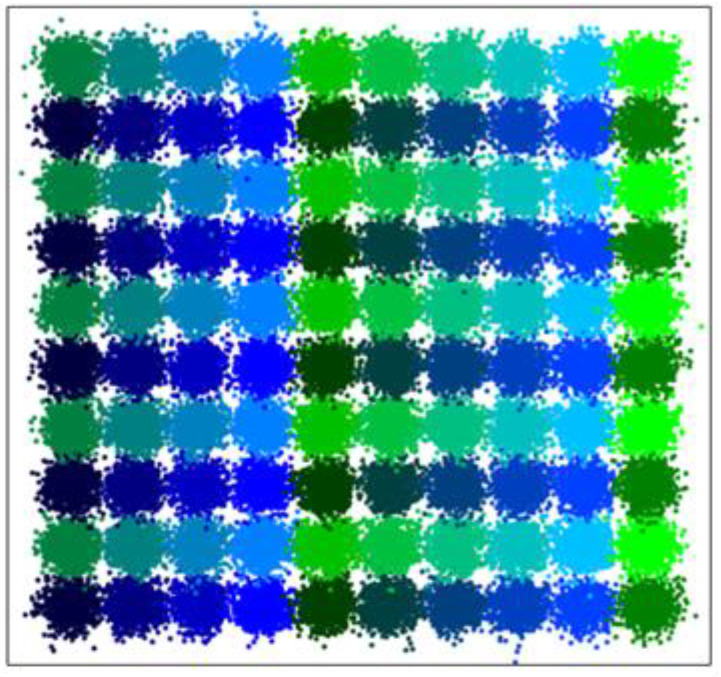
Large-scale dataset with 10^5^ points.

The ability to process large-scale datasets determines the scope of application of a clustering algorithm. This experiment is carried out on the dataset proposed in [6], as shown in Figure 6. To study the changes in clustering performance with the increasing scale of datasets, four datasets, each of which has 100 clusters, are set, and the total number of points *N* is set as 1 × 10^4^, 2 × 10^4^, 5 × 10^4^, and 10 × 10^4^, respectively. Table 5 shows the best-performing values of input parameters selected for the algorithms in Test Ⅱ. From the clustering results of large-scale datasets (Table 6), we can see that CAGS can give the best clustering accuracy for each dataset. The highest clustering accuracy of different algorithms is marked in bold, the lowest clustering accuracy is marked in italics, the highest operating efficiency is marked in bold, and the lowest operating efficiency is marked in italics. Although WaveCluster shows high efficiency in time, it obtains the worst accuracy. For WaveCluster, clustering failed due to the connection between clusters in the dataset, whereby the density difference between the cluster boundary and the cluster center is weakened. Therefore, the wavelet algorithm cannot effectively detect boundaries. To solve this problem, the points at the boundary of the clusters are set as noise to create boundaries between clusters. The processing time of *k*-means, CFSFDP, and DBSCAN increases rapidly due to their point-based clustering principle. When the total number exceeds 2 × 10^4^, CFSFDP cannot run on the computer since the processing data grows out of memory. When the total number exceeds 10 × 10^4^, CAGS outperforms other algorithms except WaveCluster.

### 4.4. Clustering High Dimensional Dataset

Generally, a dataset with more than 10 dimensions can be considered a high-dimensional dataset [31]. The high-dimensional datasets can be divided by the distribution of data points in high-dimensional space into two types: high-dimensional sparse datasets and high-dimensional dense datasets. The data points of a high-dimensional sparse dataset present a distribution in which the clusters are highly fragmented in space with a tremendous number of grids. Conversely, the data points of high-dimensional dense datasets are spatially concentrated through which some clusters could be found in the grid cells. In this paper, two high-dimensional sparse datasets selected from the benchmark datasets of the UCI machine learning repository are used for the test, called wine [30] and grammatical facial expression [31], respectively. The dataset wine has 178 instances of 3 types of wines distributed in a 13-dimensional space with at least 8192 grid cells. The dataset grammatical facial expression has 7580 instances of 5 types of expressions distributed in a 300-dimensional space with at least 2 × 10^90^ grid cells. Therefore, for grid-based clustering methods, the data points are very sparse in high-dimensional grid space. Meanwhile, other grid-based methods do not adaptively generate cells but rather generate all the cells in the grid space. This results in the grid space occupying more memory than the computer can handle. Therefore, in this section, WaveCluster and FDGB cannot be considered in the comparison. For the clustering methods that directly address the data points, such as *k*-means, DBSCAN, and CFSFDP, the clustering depends on the distances between data points by which the attributes of each dimension are averaged. This is not conducive to clustering. Table 7 shows the best-performing values of input parameters selected for the algorithms in Test Ⅲ to Test V. The results in Table 8 and Table 9 show that CAGS outperforms other algorithms in terms of overall performance.

**Figure 7:**
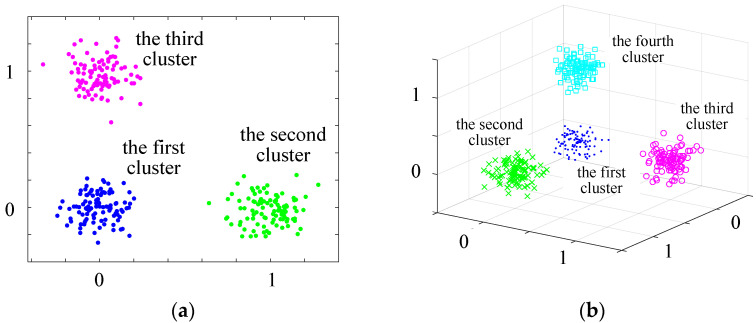
Visualization of high-dimensional dense datasets. (**a**) scatter plot of 2-dimensional dataset (**b**) scatter plot of 3-dimensional dataset.

In this paper, a simple model for generating high-dimensional dense datasets is proposed. Using this model, a *d*-dimensional dataset with *d* + 1 clusters can be generated. All clusters have Gaussian distribution with the same $\sigma^{2}$ = 0.1, as well as the (*i* + 1)th cluster has a $\mu =\langle 0,0,...,1,0,...,0\rangle$ that the *i*th coordinate value is 1 and other coordinate values are 0. Each cluster has 100 instances. For visualization, scatter plots of 2-dimensional dataset and 3-dimensional dataset are provided, as shown in Figure 7.

The results of Test V, as shown in Table 10, demonstrate that CAGS provides the best clustering accuracy when the dimensions number d are 10, 20, 30, and 40, respectively. In this test, CAGS achieves clustering by generating a few grids, so the processing speed is very fast. Through Test Ⅲ, Test Ⅳ, and Test V, the effectiveness of CAGS can be found in both high dimensional sparse datasets and dense datasets.

### 4.5. Clustering Dataset with Arbitrary Shapes

**Figure 8:**
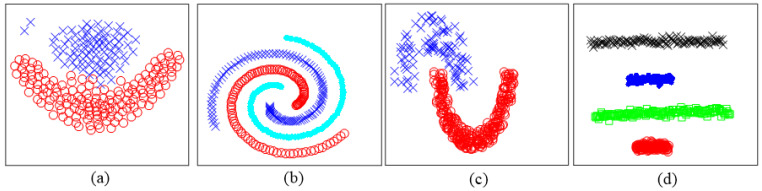
Distribution of datasets with complex shapes. (**a**) Flame (**b**) 3-spiral (**c**) Jain (**d**) Sticks (Test VI to Test IX).

The datasets with arbitrary shapes are common to be seen in the pixel distribution, and the clustering of these datasets contributes to image processing. Four typical datasets with arbitrary shapes (as shown in Figure 8) called Flame [32], 3-spiral [33], Jain [34], and Sticks [33] are chosen to test the clustering performance. Table 11 shows the best-performing values of input parameters selected for the algorithms in Test VI to Test IX. The results in Table 12 demonstrate that CAGS can successfully process the datasets with different kinds of complex shapes. Obviously, *k*-means are ineffective when processing spiral-shaped data. For DBSCAN, clustering failed due to the connection between two clusters in the dataset Flame. It is notable that CFSFDP performs poorly on the dataset Sticks because the density contrast between different clusters is too large. In addition, despite extensive attempts, we have not yet found the optimal parameters for FDGB to successfully cluster datasets of Test VI and Test VII, as mentioned in [21].

### 4.6. Clustering Dataset with Large Differences in Density between Classes

**Figure 9:**
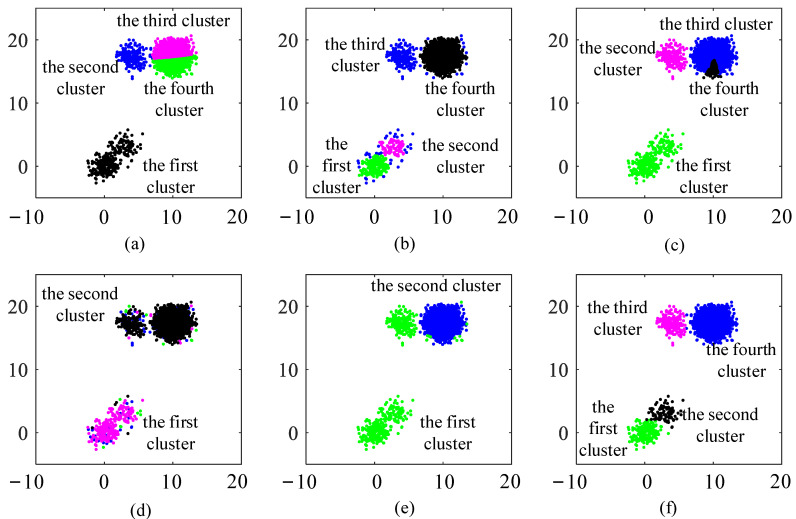
Clustering results of the dataset with large differences in density between classes. (**a**–**f**) Clustering results obtained using *k*-means, DBSCAN, CFSFDP, WaveCluster, FDGB, and CAGS, respectively.

If clusters in a dataset are of significant difference in density, clusters with high density will have an impact on clusters with low density in the clustering process. Many algorithms are ineffective for these kinds of datasets. In this section, we propose a synthetic dataset containing four clusters that have 100, 50, 200, and 5000 points from bottom left to top right, respectively. All clusters have Gaussian distribution, as well as the first cluster has $\mu =\langle 0,0\rangle$ and $\sigma^{2}$ = 3, the second cluster has $\mu =\langle 3,3\rangle$ and $\sigma^{2}$ = 3, the third cluster has $\mu =\langle 4,17.3\rangle$ and $\sigma^{2}$ = 2, and the fourth cluster has $\mu =\langle 10,17.3\rangle$ and $\sigma^{2}$ = 3. Table 13 shows the best-performing values of input parameters selected for the algorithms in Test X. From the clustering results listed in Figure 9; it is clear that only CAGS and DBSCAN could recognize all clusters successfully. However, DBSCAN confronts problems when classifying the bottom left two clusters due to the small distance between them. For *k*-means and CFSFDP, the correct number of clusters is provided to them. However, clustering performance is undesirable, which can be attributed to the distance-based clustering strategy. For FDGB, clustering performance is impacted by the large density difference. Specifically, when looking for mountain ridges, all of them appeared in the cluster in the upper right corner, resulting in clusters with low density being undetectable.

### 4.7. Clustering Dataset with High Overlap between Classes

**Figure 10:**
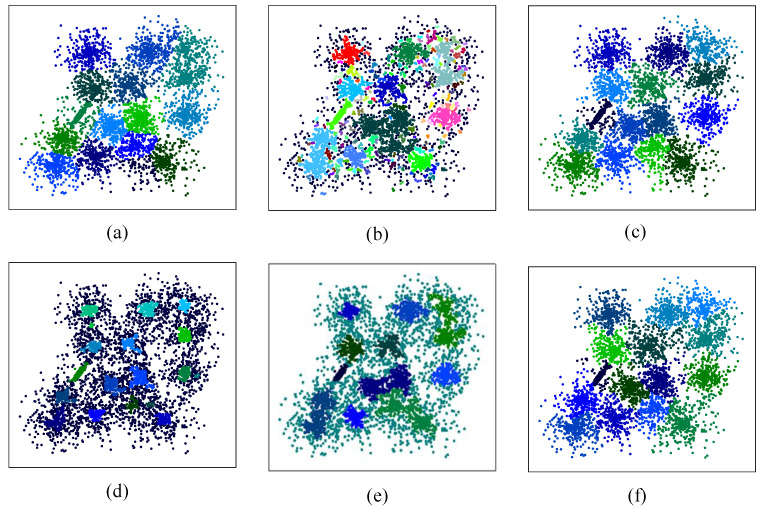
Distribution and clustering results of the dataset with high overlap between classes. (**a**–**f**) Clustering results obtained using *k*-means, DBSCAN, CFSFDP, WaveCluster, FDGB, and CAGS, respectively.

In this section, a dataset [35] with 15 strongly overlapping clusters is selected to test clustering performance on datasets with high overlap between classes. Table 14 shows the best-performing values of input parameters selected for the algorithms in Test XI. The original distribution of the dataset and the clustering results are shown in Figure 10a–f. The results show that only CAGS and CFSFDP could successfully identify all 15 clusters. For DBSCAN, WaveCluster, and FDGB, clusters can only be identified when the halo data are processed as noise data. Thus, they are ineffective for this kind of dataset. For *k*-means, only when the input number of clusters is 14 can each cluster be well identified.

## 5. Conclusions

In this paper, a new grid-based clustering method called CAGS is proposed. Our algorithm has two main innovations compared to current grid-based clustering methods. Firstly, an adaptive grid-space constructing strategy is established to generate the minimum cell set that covers all data points. Thus, it can prevent the sharp rise of cell numbers when the range of point distribution or the dimension number of the dataset is too large. Secondly, a clustering strategy based on adjacent grid searching is constructed to expand the clusters with arbitrary shapes. CAGS can recognize noise cells and peripheral cells at different levels based on two adaptive parameters $f_{N}$ and $f_{H}$, which ensures the successful performance for clustering the dataset with noise and the dataset with high overlap between classes. The CAGS is then tested using six different types of datasets, as mentioned in the introduction. Several typical clustering methods, such as *k*-means, DBSCAN, CFSFDP, WaveCluster, and FDGB, are used for comparison. The results show that CAGS can successfully deal with all the above types of datasets, demonstrating the satisfactory robustness of CAGS for future practical applications. However, the proposed algorithm will encounter a challenge if a dataset with a small number of data points has complex shapes. In this case, the cell density is very low, so the location of a single data point significantly impacts the cell. Thus, such datasets often require specific grid sizes to achieve good clustering. In future work, an improved grid space construction strategy that determines the cell size based on data distribution will be adopted to address this problem. In addition, we will use CAGS to resolve the issues in image processing, unsupervised pattern recognition, and big data analysis.

## Figures and Tables

**Table 1:** Meshing result of a 2-dimensional dataset.

Cell Number	Location	Density	Member
1	<1, 1>	1	15
2	<2, 1>	3	1, 3, 7
3	<3, 1>	1	12
5	<1, 2>	1	13
6	<2, 2>	2	4,6
7	<3, 2>	4	10, 17, 18, 19
8	<4, 2>	2	14, 20
11	<3, 3>	2	2, 5
12	<4, 3>	1	11
14	<2, 4>	1	16
15	<3, 4>	1	9
16	<4, 4>	1	8

**Table 3:** Best performing values of input parameters selected for the algorithms in Test Ⅰ.

Algorithm	SNR = 100%	SNR = 90%	SNR = 70%	SNR = 50%
*k*-means				
*k*	4	4	4	4
DBSCAN				
*k*	10	6	8	4
*Eps*	Default	Default	Default	Default
CFSFDP				
*k*	4	4	4	4
WaveCluster				
*weights*	Default	Default	Default	Default
*num_cells*	250	250	250	250
*densitythreshold*	0%	5%	25%	40%
*level*	1	1	1	1
FDGB				
*no_grid*	20	20	20	20
*thre_grid_length*	1.1	1.1	1.1	1.1
*cutoff_factor*	0.21	0.21	0.21	0.21
*noise_thre*	0	1	1.5	2.5
CAGS				
*Nfac*	0	0.35	0.7	1.6
*Rfac*	0.3	0.3	0.3	0.3
*Hfac*	0.5	0.5	0.5	0.5
*Mfac*	0	0	0	0

**Table 4:** Clustering results of datasets with different levels of noise (Test Ⅰ).

Algorithm	SNR = 100%	SNR = 90%	SNR = 70%	SNR = 50%
*PUR*				
*k*-means	**1.00**	0.89	0.69	0.49
DBSCAN	0.99	**0.97**	0.89	0.72
CFSFDP	**1.00**	0.93	0.90	0.88
WaveCluster	*0.80*	*0.75*	*0.55*	*0.34*
FDGB	0.95	0.87	0.68	0.49
CAGS	**1.00**	0.96	**0.92**	**0.88**
*CSM*				
*k*-means	**1.00**	*0.80*	*0.71*	*0.58*
DBSCAN	0.83	0.95	0.90	**0.90**
CFSFDP	**1.00**	0.87	0.90	0.88
WaveCluster	*0.73*	*0.80*	0.77	0.70
FDGB	0.97	0.95	**0.92**	0.87
CAGS	**1.00**	**0.96**	**0.92**	0.88
*NMI*				
*k*-means	0.98	0.83	*0.65*	*0.49*
DBSCAN	0.98	**0.92**	0.80	0.64
CFSFDP	**1.00**	0.86	0.80	0.74
WaveCluster	*0.75*	*0.77*	0.74	0.68
FDGB	0.92	0.90	0.80	0.76
CAGS	**1.00**	**0.92**	**0.81**	**0.77**
*CluCE*				
*k*-means	0.02	*0.19*	*0.34*	*0.39*
DBSCAN	0.01	**0.07**	0.17	**0.10**
CFSFDP	**0.00**	0.15	0.17	0.18
WaveCluster	*0.17*	0.14	**0.14**	0.14
FDGB	0.04	**0.07**	0.13	0.15
CAGS	**0.00**	**0.07**	0.17	0.14
*ClaCE*				
*k*-means	0.02	0.11	*0.29*	*0.46*
DBSCAN	0.03	**0.07**	0.20	0.32
CFSFDP	**0.00**	0.11	0.21	0.26
WaveCluster	*0.18*	*0.12*	**0.13**	**0.16**
FDGB	0.10	0.11	0.18	0.24
CAGS	**0.00**	0.08	0.19	0.17

**Table 5:** Best performing values of input parameters selected for the algorithms in Test Ⅱ.

Algorithm	N = 1 × 10^4^	N = 2 × 10^4^	N = 5 × 10^4^	N = 10 × 10^4^
*k*-means				
*k*	100	100	100	100
DBSCAN				
*k*	1	1	1	1
*Eps*	Default	Default	Default	Default
CFSFDP				
*k*	100	/	/	/
WaveCluster				
*weights*	Default	Default	Default	Default
*num_cells*	110	110	110	110
*densitythreshold*	60%	60%	60%	60%
*level*	2	2	2	2
FDGB				
*no_grid*	30	30	30	30
*thre_grid_length*	1.5	1.5	1.5	1.5
*cutoff_factor*	0.5	0.5	0.5	0.5
*noise_thre*	1	1	1	1
CAGS				
*Nfac*	0	0	0	0
*Rfac*	0.5	2^(−0.5)/2	5^(−0.5)/2	10^(−0.5)/2
*Hfac*	1	1	1	1
*Mfac*	0	0	0	0

**Table 6:** Clustering results of large-scale datasets (Test Ⅱ).

Algorithm	N = 1 × 10^4^	N = 2 × 10^4^	N = 5 × 10^4^	N = 10 × 10^4^
*PUR*				
*k*-means	0.87	0.88	0.91	0.86
DBSCAN	0.84	0.87	0.86	0.85
CFSFDP	*0.43*	/	/	/
WaveCluster	0.78	0.81	0.81	0.82
FDGB	0.91	0.92	0.91	0.92
CAGS	**0.95**	**0.96**	**0.97**	**0.97**
*CSM*				
*k*-means	0.85	0.87	0.87	0.87
DBSCAN	0.86	0.92	0.91	0.90
CFSFDP	*0.57*	*/*	*/*	*/*
WaveCluster	0.79	0.81	0.82	0.82
FDGB	0.92	0.93	0.93	0.94
CAGS	**0.96**	**0.97**	**0.97**	**0.97**
*NMI*				
*k*-means	0.93	0.94	0.94	0.93
DBSCAN	0.90	0.90	0.89	0.87
CFSFDP	0.85	/	/	/
WaveCluster	*0.76*	*0.77*	*0.78*	*0.78*
FDGB	0.94	0.94	0.93	0.93
CAGS	**0.96**	**0.96**	**0.96**	**0.96**
*CluCE*				
*k*-means	0.08	0.07	0.07	0.07
DBSCAN	0.12	0.10	0.11	0.12
CFSFDP	0.21	/	/	/
WaveCluster	*0.31*	*0.30*	*0.29*	*0.29*
FDGB	0.07	0.07	0.07	0.08
CAGS	**0.04**	**0.04**	**0.04**	**0.04**
*ClaCE*				
*k*-means	0.06	0.06	0.06	0.06
DBSCAN	0.07	0.08	0.09	0.09
CFSFDP	0.07	/	/	/
WaveCluster	*0.13*	*0.14*	*0.14*	*0.14*
FDGB	0.05	0.06	0.06	0.07
CAGS	**0.04**	**0.04**	**0.04**	**0.04**
Time (s)				
*k*-means	0.66	1.37	4.02	19.35
DBSCAN	2.39	8.77	*52.6*	*456*
CFSFDP	*13.86*	/	/	/
WaveCluster	**0.009**	**0.006**	**0.011**	**0.021**
FDGB	0.11	0.10	0.17	0.31
CAGS	11.0	*13.2*	15.5	18.5

**Table 7:** Best performing values of input parameters selected for the algorithms in Test III to Test V.

Algorithm	Test III	Test IV	Test V (Dimension Number)			
			10	20	30	40
*k*-means						
*k*	3	5	11	21	31	41
DBSCAN						
*k*	2	4	10	10	10	10
*Eps*	Default	Default	Default	Default	Default	Default
CFSFDP						
*k*	3	5	11	21	31	41
CAGS						
*Nfac*	0	0	0	0	0	0
*Rfac*	1.5	5	0.5	0.5	0.5	0.5
*Hfac*	0	0	0	0	0	0
*Mfac*	0.4	0.5	0	0	0	0

**Table 8:** Clustering results of high dimensional sparse dataset: wine (Test III).

Algorithm	PUR	CSM	NMI	CluCE	ClaCE	Time
*k*-means	0.70	0.70	**0.43**	**0.56**	0.56	0.09
DBSCAN	*0.02*	*0.37*	*0.05*	*0.77*	**0.05**	**0.02**
CFSFDP	0.71	0.71	0.42	0.57	0.58	0.26
CAGS	**0.74**	**0.74**	0.36	0.64	*0.63*	*0.63*

**Table 9:** Clustering results of high dimensional sparse dataset: grammatical facial expression (Test IV).

Algorithm	PUR	CSM	NMI	CluCE	ClaCE	Time
*k*-means	0.40	0.43	0.18	0.81	*0.66*	**0.08**
DBSCAN	*0.27*	*0.33*	*err*	*0.98*	**0**	*203*
CFSFDP	0.38	0.38	0.18	0.84	0.44	3.15
CAGS	**0.62**	**0.64**	**0.56**	**0.54**	**0.19**	0.54

**Table 10:** Clustering results of high dimensional dense datasets (Test V).

Algorithm	Dimension Number	PUR	CSM	NMI	CluCE	ClaCE	Time
k-means	10	0.74	0.82	0.91	0.13	0.05	0.03
	20	0.61	0.68	0.86	0.21	0.06	0.03
	30	0.86	0.91	0.97	0.04	0.02	0.08
	40	0.56	0.64	0.86	0.21	0.06	0.08
DBSCAN	10	0.09	0.16	err	1	**0**	0.12
	20	0.05	0.09	err	1	**0**	1.42
	30	0.03	0.06	err	1	**0**	4.81
	40	0.02	0.05	err	1	**0**	27.5
CFSFDP	10	**1**	**1**	**1**	**0**	**0**	0.13
	20	0.72	0.75	0.87	0.21	0.03	0.55
	30	0.55	0.65	0.86	0.23	0.03	1.37
	40	0.54	0.63	0.87	0.23	0.02	2.58
CAGS	10	**1**	**1**	**1**	**0**	**0**	**0.01**
	20	**1**	**1**	**1**	**0**	**0**	**0.01**
	30	**1**	**1**	**1**	**0**	**0**	**0.01**
	40	**1**	**1**	**1**	**0**	**0**	**0.02**

**Table 11:** Best performing values of input parameters selected for the algorithms in Test VI to Test IX.

Algorithm	Flame	3-Spiral	Jain	Sticks
*k*-means				
*k*	2	3	2	4
DBSCAN				
*k*	2	2	2	2
*Eps*	Default	Default	Default	Default
CFSFDP				
*k*	2	3	2	4
WaveCluster				
*weights*	Default	Default	Default	Default
*num_cells*	100	100	100	100
*densitythreshold*	0%	20%	30%	30%
*level*	1	2	2	2
FDGB				
*no_grid*	20	20	20	10
*thre_grid_length*	1.1	1.1	1.1	1.1
*cutoff_factor*	0.95	0.21	0.21	0.21
*noise_thre*	0	0	0	0
CAGS				
*Nfac*	0	0	0	0
*Rfac*	0.5	1.5	0.8	0.5
*Hfac*	0.9	0	1.2	0
*Mfac*	0.1	0	0	0

**Table 12:** Clustering results of the dataset with arbitrary shapes (Test VI to Test IX).

Algorithm	Flame	3-Spiral	Jain	Sticks
PUR				
*k*-means	0.84	*0.35*	*0.79*	0.75
DBSCAN	*0.44*	**1**	0.93	**1**
CFSFDP	0.79	**1**	0.86	*0.41*
WaveCluster	0.59	**1**	0.89	**1**
FDGB	0.64	0.60	0.91	0.99
CAGS	0.98	**1**	**1**	**1**
CSM				
*k*-means	0.84	0.35	0.77	0.75
DBSCAN	0.42	**1**	0.61	**1**
CFSFDP	0.79	**1**	0.84	0.50
WaveCluster	0.75	**1**	0.87	**1**
FDGB	0.67	0.75	0.90	0.99
CAGS	**0.98**	**1**	**1**	**1**
NMI				
*k*-means	0.43	**0.00**	0.37	0.70
DBSCAN	0.46	**1**	0.87	**1**
CFSFDP	0.41	**1**	0.51	0.42
WaveCluster	0.55	**1**	0.76	**1**
FDGB	0.65	0.78	0.90	0.95
CAGS	**0.87**	**1**	**1**	**1**
CluCE				
*k*-means	0.53	1	0.49	0.31
DBSCAN	*0.61*	**0**	0.13	**0**
CFSFDP	0.12	**0**	**0**	**0**
WaveCluster	**0.03**	**0**	**0**	**0**
FDGB	0.59	**0**	**0**	**0**
CAGS	0.12	**0**	**0**	**0**
ClaCE				
*k*-means	0.58	1	0.66	0.26
DBSCAN	0.13	**0**	**0**	**0**
CFSFDP	*0.59*	**0**	0.52	0.49
WaveCluster	0.43	**0**	0.15	0
FDGB	0.54	0.37	0.14	**0**
CAGS	**0.11**	**0**	**0**	**0**

**Table 13:** Best performing values of input parameters selected for the algorithms in Test X.

Algorithm	Parameters			
*k*-means	*k*			
	4			
DBSCAN	*k*		*Eps*	
	9		Default	
CFSFDP	*k*			
	4			
WaveCluster	*weights*	*num_cells*	*densitythreshold*	*level*
	Default	100	50%	2
FDGB	*no_grid*	*thre_grid_length*	*cutoff_factor*	*noise_thre*
	10	2	0.21	1.5
CAGS	*Nfac*	*Rfac*	*Hfac*	*Mfac*
	0	0.2	0.2	0

**Table 14:** Best performing values of input parameters selected for the algorithms in Test XI.

Algorithm	Parameters			
*k*-means	*k*			
	15			
DBSCAN	*k*		*Eps*	
	2		Default	
CFSFDP	*k*			
	15			
WaveCluster	*weights*	*num_cells*	*densitythreshold*	*level*
	Default	50	90%	1
FDGB	*no_grid*	*thre_grid_length*	*cutoff_factor*	*noise_thre*
	40	1.5	0.21	1.5
CAGS	*Nfac*	*Rfac*	*Hfac*	*Mfac*
	0	0.7	6	0.1

## Data Availability

The data, code, and other materials can be made available on request.

## References

[1] Kaufman L., Rousseeuw P.J. (2009). Finding Groups in Data: An Iintroduction to Cluster Analysis.

[2] Martín Merino M., López Rivero A.J., Alonso V., Vallejo M., Ferreras A. (2022). A Clustering Algorithm Based on an Ensemble of Dissimilarities: An Application in the Bioinformatics Domain. Int. J. Interact. Multimed. Artif. Intell..

[3] Seal A., Herrera Viedma E. (2021). Performance and convergence analysis of modified C-means using jeffreys-divergence for clustering. Int. J. Interact. Multimed. Artif. Intell..

[4] MacQueen J. Some methods for classification and analysis of multivariate observations. Proceedings of the Fifth Berkeley Symposium on Mathematical Statistics and Probability.

[5] Bezdek J.C., Ehrlich R., Full W. (1984). FCM: The fuzzy c-means clustering algorithm. Comput. Geosci..

[6] Ester M., Kriegel H.P., Sander J., Xu X. (1996). A density-based algorithm for discovering clusters in large spatial databases with noise. Kdd.

[7] Zhang T., Ramakrishnan R., Livny M. (1996). BIRCH: An efficient data clustering method for very large databases. ACM Sigmod Rec..

[8] Liu B., Xia Y., Yu P.S. Clustering through decision tree construction. Proceedings of the Ninth International Conference on Information and Knowledge Management.

[9] Xie W.B., Liu Z., Srivastava J. (2021). Hierarchical clustering by aggregating representatives in sub-minimum-spanning-trees. arXiv.

[10] Xie W.B., Liu Z., Das D., Chen B., Srivastava J. (2023). Scalable clustering by aggregating representatives in hierarchical groups. Pattern Recognit..

[11] Rodriguez A., Laio A. (2014). Clustering by fast search and find of density peaks. Science.

[12] Zhao Y., Wang H., Pei J. (2019). Deep non-negative matrix factorization architecture based on underlying basis images learning. IEEE Trans. Pattern Anal. Mach. Intell..

[13] Wang D., Li T., Deng P., Wang H., Zhang P. (2022). Dual graph-regularized sparse concept factorization for clustering. Inf. Sci..

[14] Filippone M., Camastra F., Masulli F., Rovetta S. (2008). A survey of kernel and spectral methods for clustering. Pattern Recognit..

[15] Schikuta E. Grid-clustering: An efficient hierarchical clustering method for very large data sets. Proceedings of the 13th International Conference on Pattern Recognition.

[16] Wang W., Yang J., Muntz R. STING: A statistical information grid approach to spatial data mining. Proceedings of the VLDB.

[17] Sheikholeslami G., Chatterjee S., Zhang A. (2000). WaveCluster: A wavelet-based clustering approach for spatial data in very large databases. VLDB J..

[18] Agrawal R., Gehrke J., Gunopulos D., Raghavan P. Automatic subspace clustering of high dimensional data for data mining applications. Proceedings of the 1998 ACM SIGMOD International Conference on Management of Data.

[19] Hinneburg A., Keim D.A. Optimal grid-clustering: Towards breaking the curse of dimensionality in high-dimensional clustering. Proceedings of the 25th International Conference on Very Large Data Bases (VLDB).

[20] Yanchang Z., Junde S. GDILC: A grid-based density-isoline clustering algorithm. Proceedings of the 2001 International Conferences on Info-Tech and Info-Net.

[21] Wu B., Wilamowski B.M. (2016). A fast density and grid-based clustering method for data with arbitrary shapes and noise. IEEE Trans. Ind. Inform..

[22] Du M., Wu F. (2022). Grid-Based Clustering Using Boundary Detection. Entropy.

[23] Starczewski A., Scherer M.M., Książek W., Dębski M., Wang L. (2021). A novel grid-based clustering algorithm. J. Artif. Intell. Soft Comput. Res..

[24] Yan Y., Sun Z., Mahmood A., Xu F., Dong Z., Sheng Q.Z. (2022). Achieving Differential Privacy Publishing of Location-Based Statistical Data Using Grid Clustering. ISPRS Int. J. Geo-Inf..

[25] Chen J., Sackey S.H., Ansere J.A., Zhang X., Ayush A. (2022). A Neighborhood Grid Clustering Algorithm for Solving Localization Problem in WSN Using Genetic Algorithm. Comput. Intell. Neurosci..

[26] Wang X., Zhang Z., Luo Y. (2022). Clustering Methods Based on Stay Points and Grid Density for Hotspot Detection. ISPRS Int. J. Geo-Inf..

[27] Song M., Zhang L. Comparison of cluster representations from partial second-to full fourth-order cross moments for data stream clustering. Proceedings of the 2008 8th IEEE International Conference on Data Mining.

[28] Zhang H., Ho T.B., Zhang Y., Lin M.S. (2006). Unsupervised feature extraction for time series clustering using orthogonal wavelet transform. Informatica.

[29] Strehl A., Ghosh J. (2002). Cluster ensembles—A knowledge reuse framework for combining multiple partitions. J. Mach. Learn. Res..

[30] UCI Machine Learning Repository. http://archive.ics.uci.edu/ml/index.php.

[31] De Almeida Freitas F., Peres S.M., de Moraes Lima C.A., Barbosa F.V. Grammatical facial expressions recognition with machine learning. Proceedings of the 27th International Flairs Conferenc.

[32] Fu L., Medico E. (2007). FLAME, a novel fuzzy clustering method for the analysis of DNA microarray data. BMC Bioinform..

[33] Chang H., Yeung D.Y. (2008). Robust path-based spectral clustering. Pattern Recognit..

[34] Jain A.K., Law M.H. (2005). Data clustering: A user’s dilemma. Pattern Recognition and Machine Intelligence: First International Conference, PReMI 2005, Kolkata, India, 20–22 December 2005.

[35] Fränti P., Virmajoki O. (2006). Iterative shrinking method for clustering problems. Pattern Recognit..

